# Magnetic Properties of the Soft Magnetic Composites Prepared Using Mixtures of Carbonyl Iron, FeSiCr, and FeSiAl Alloy Powders

**DOI:** 10.3390/ma16176033

**Published:** 2023-09-02

**Authors:** Hsing-I Hsiang, Liang-Fang Fan

**Affiliations:** Department of Resources Engineering, National Cheng Kung University, Tainan 70101, Taiwan

**Keywords:** soft magnetic composites, carbonyl iron powder, FeSiCr alloy powder, nanocrystalline magnetic powder

## Abstract

The effect of carbonyl iron powder, FeSiCr alloy powder, and annealed FeSiAl alloy powder, both individually and in binary combinations, on the density, microstructure, and magnetic properties (including permeability and power loss) of inductors manufactured by molding compaction was investigated in this study. The investigation demonstrates that hysteresis loss dominates power loss in the tested frequency range. Due to higher compacted density and reduced coercivity, adding 50% carbonyl iron powder to annealed powder resulted in the lowest hysteresis loss, allowing for domain wall movement. On the other hand, adding 50% FeSiCr alloy powder to annealed powder resulted in higher hysteresis loss due to impurity components hindering domain wall motion. Due to extreme plastic deformation, the carbonyl iron powder and FeSiCr alloy powder combinations displayed the most significant hysteresis loss. Eddy current loss followed the same trends as hysteresis loss in the mixtures. This study provides important insights for refining the soft magnetic composite design to obtain higher magnetic performance, while minimizing power loss.

## 1. Introduction

Soft magnetic composites (SMCs) offer several advantages in power systems and magnetic components for energy storage and transmission. Low core loss at high operating frequencies, steady permeability with rising test frequency and bias current, reduced eddy current losses, increased frequency stability of permeability, and improved DC bias performance are among the benefits [1,2,3].

Inductors are key components in electronic and electrical systems, where they convert and store energy. Magnetic properties, particularly permeability and power loss, significantly impact the performance of inductors. Power molding inductors are commonly employed for electromagnetic storage or noise filtering in power modules. They blend soft magnetic composites with coil winding to build a monolithic structure. The power module in automobile power modules frequently runs at high frequencies and in difficult situations (e.g., high temperature and humidity). Power molding inductors’ power loss mainly comprises hysteresis loss and eddy current [4]. At low frequencies, hysteresis loss dominates magnetic core loss. The alloy’s coercivity and compaction density have a significant impact on hysteresis loss [5]. Eddy current loss grows rapidly with frequency and is classified into two types: inter-particle eddy current loss and intra-particle eddy current loss [1]. The resistivity and particle size of the alloy powders are the primary determinants of intra-particle eddy current loss [6]. Carbonyl iron powder (CIP) is a promising candidate for efficient inductor cores due to its high permeability and minimal power loss [7]. FeSiCr alloy powder, on the other hand, offers a balance between permeability and power loss, providing adaptability in inductor design [8]. Compared to typical polycrystalline magnetic alloys, nanocrystalline and amorphous magnetic alloy materials exhibit higher permeability, lower coercivity, and lower power loss [9]. Amorphous and nanocrystalline Fe-based alloys have gained favor as magnetic materials for a new generation of power molding inductors used in high-frequency electronic applications due to their superior soft magnetic properties [10]. On the other hand, amorphous and nanocrystalline Fe-based alloy powders have significant brittleness and hardness, making full-density compacting difficult. Carbonyl iron powder (CIP) has many benefits, including low cost and high saturation magnetization [11]. According to Hsiang et al. [12,13], introducing CIP into FeSiCr alloy powders and amorphous alloy powder can increase the compaction density of molding coils due to significant plastic deformation during pressing, resulting in increased inductance. However, a detailed evaluation of the magnetic characteristics of soft magnetic composites made with individual and binary mixes of carbonyl iron powder, FeSiCr alloy powder, and nanocrystalline alloy powder has yet to be performed.

The effects of individual and binary magnetic powder combinations on the density, microstructure, and magnetic characteristics of soft magnetic composites are investigated in this study. Carbonyl iron powder, FeSiCr alloy powder, and nanocrystalline powder are among the magnetic powders under investigation. Understanding compaction capabilities is crucial for attaining high-density packing and limiting porosity, directly impacting the magnetic characteristics and overall performance of powder-molded inductors. During the fabrication process, the compaction behaviors of each powder were examined. Furthermore, we explored the advantages of employing binary mixtures of these magnetic powders in powder molding. Combining different powders makes it possible to tailor the inductor’s properties to meet specific application requirements. The variations in density, microstructure, and resulting magnetic properties in these mixtures provide valuable insights for optimizing inductor design and achieving superior magnetic performance with reduced power loss.

The findings from this study have significant implications for the design and development of powder-molded inductors, offering insights into enhancing magnetic performance, increasing energy efficiency, and reducing power loss. The research outcomes are expected to advance the field of magnetic materials and contribute to the design of high-performance inductor systems for various electronic and electrical applications.

## 2. Experimental Procedure

The raw materials for this study were FeSiCr alloy powder (Epson Atmix Co., Ltd., Hachinohe, Japan), carbonyl iron powder (Jiangxi Yuean Advanced Materials Co., Ltd., Dayu, China), and FeSiAl annealed powder (Shenzhen POCO Magnetic Co., Ltd., Shenzhen, China). The FeSiAl annealed powder is produced by air-atomization (pressure range of 0.7 to 5 MPa) and then undergoes a specialized annealing procedure at 550 °C. The annealing process promotes the formation of a nanocrystalline structure within the powder, significantly reducing magnetic loss [13]. This study applied an insulating iron phosphate layer onto the carbonyl iron powder surface, employing a wet-chemical technique described in our previous study [14]. A total of 100 g of magnetic powder was combined with 50 mL of acetone and subjected to ultrasonication for 10 min. Subsequently, 2 wt% of phosphoric acid was introduced and stirred at a speed of 250 rpm for 30 min [14]. Eventually, the excess acetone was entirely evaporated, and the resulting mixture was dried at a temperature of 80 °C for 2 h, followed by sieving through a 140-mesh screen to acquire the phosphated magnetic powders.

A binary mixture was formed by blending the magnetic powder at a powder ratio of 50 wt% using a V-cone apparatus. The phosphated magnetic powder was blended with 3 wt% phenolic resin, utilizing acetone as the solvent. After drying and granulation through a 40-mesh screen, the granulated powders were dry-pressed at 600 MPa to create toroidal bodies with an outer diameter of 12.85 mm and an inner diameter of 7.75 mm. These bodies were subsequently cured at 150 °C for 2 h.

The tapped densities were measured using a tapped density tester (AT-2000, Quantachrome, Boynton Beach, FL, USA). The true densities were measured using a helium pycnometer (Accupyc 1330, Micromeritics, Norcross, GA, USA). The relative compacted densities were calculated using the compacted density and theoretical density ratios of the samples after compaction. A scanning electron microscope (SEM) from TEMIC EM200S, Hsinchu, Taiwan, was used to examine the morphology of the magnetic powders and compact microstructures. The initial permeabilities of the toroidal bodies were analyzed using a precision impedance analyzer (WK6500B, Wayne Kerr Electronics Co., Ltd., London, UK) under a voltage setting of 0.25 V and a frequency of 100 kHz. A B-H analyzer (SY-8218) from IWATSU Electronics Co., Ltd., Tokyo, Japan, was employed to calculate the coercivities and power losses of the toroidal bodies. 

## 3. Results and Discussion

### 3.1. Carbonyl Iron Powder, FeSiCr Alloy Powder, and Annealed Alloy Powder

Table 1 displays the particle size distribution, powder shape, true density, tap density, and chemical composition of magnetic powders. The FeSiCr alloy powder has an irregular morphology, whereas the carbonyl iron and annealed powders are nearly spherical. The FeSiCr alloy, carbonyl iron, and annealed powder have mean particle sizes of 9.4, 4.4, and 9.1 µm, respectively. Table 2 compares the compacted density and initial permeability of carbonyl iron powder, FeSiCr alloy powder, and annealed alloy powder. Carbonyl iron powder had the highest compacted density (92.4%), while FeSiCr alloy and annealed powder had relative densities of 87.0 and 79.2%, respectively. Because of the lower content of alloy elements, the hardness of carbonyl iron powder is much lower than that of FeSiCr alloy powder and annealed powder, resulting in the highest compacted density under 600 MPa pressure. Even though the better flowability of the spherical annealed powder increases tap density, the lowest relative compacted density was obtained due to lower powder deformation caused by the abundance of grain boundaries, which hinders dislocation motion [15]. It does not deform when compressed, preventing the powder from producing mechanical locking during molding. As a result, when the pressure is released, the spherical powder produces a spring-back effect, decreasing relative density. As a result, nanocrystalline powders are rarely used alone for molding inductors; instead, they must be combined with other iron-based metal powders. 

**Figure 1:**
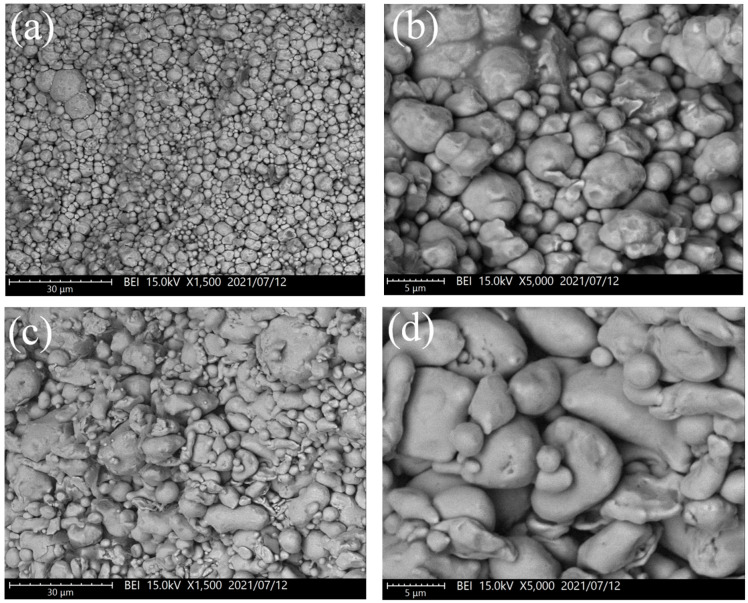

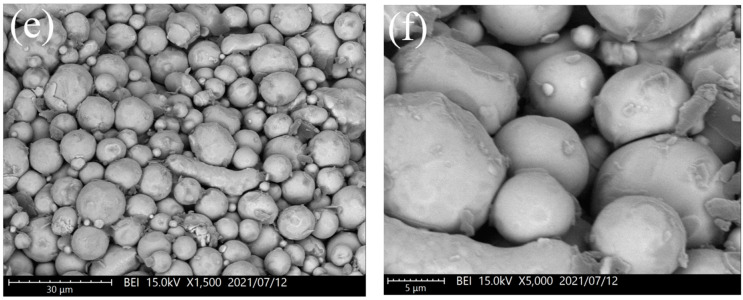
SEM images of the samples compacted at 600 MPa: (**a**,**b**) carbonyl iron powder, (**c**,**d**) FeSiCr alloy powder, and (**e**,**f**) annealed powder.

The SEM images of the compacted samples are shown in Figure 1. The compacted carbonyl iron powder had a denser microstructure because of the severe plastic deformation. On the other hand, the annealed powder remained nearly spherical after compaction with minor shape deformation, increasing porosity. This is consistent with the result of relative compacted density (Table 2). Despite carbonyl iron powder having the highest compacted density, the FeSiCr alloy powder had the highest magnetic permeability. During the molding process, carbonyl iron powder produced many defects inside the particles, causing large plastic deformation and reducing the magnetic permeability of the powder [8]; so, the magnetic permeability of the powder was lower than that of the FeSiCr alloy powder. The magnetic permeability of compacted annealed powder was significantly lower than that of FeSiCr alloy powder due to its low relative density of 79.2%.

**Figure 2:**
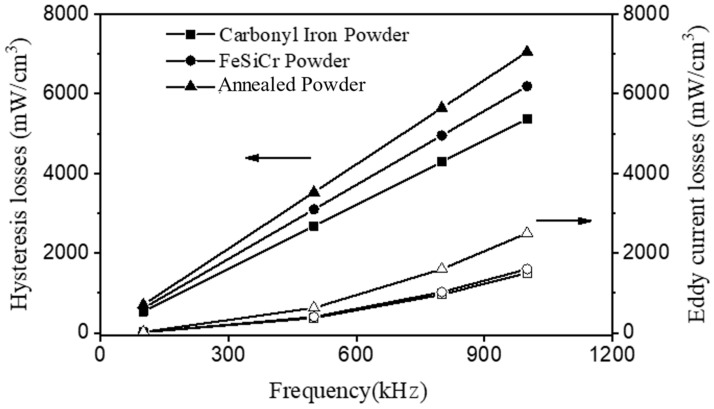
Dependence of the eddy current loss (P_e_) and hysteresis loss (P_h_) on the measurement frequency for the toroidal cores compacted at 600 MPa.

The total core loss of the toroidal core is composed of the hysteresis loss (P_h_), the eddy current loss (P_e_), and the residual loss (P_r_), the last of which is essential only at very low induction levels and at very high frequencies and is, thus, ignorable in this investigation. As a result, the P_cv_ may be separated using the following equation [4]:

P_cv_ = P_e_ + P_h_ = K_e_ × f^2^ + K_h_ × f (1)

where K_e_, f, and K_h_ are the eddy current loss factor, frequency, and hysteresis loss coefficient, respectively. Figure 2 shows the eddy current loss (P_e_) and hysteresis loss (P_h_) dependence on the measurement frequency for the toroidal cores. In this frequency range, the power losses of these three powders were still dominated by hysteresis loss, with carbonyl iron powder having the lowest, alloy powder having the next highest, and annealed powder having the highest. The magnetic losses of the toroidal cores are summarized in Table 3. Because hysteresis loss is primarily caused by the obstruction of magnetic domain wall motion and magnetic domain rotation [16], carbonyl iron powder had the lowest hysteresis loss due to the lowest impurity elements, the highest compacted density (the smallest porosity), and the lowest coercivity. At 1 MHz, the hysteresis losses of carbonyl iron powder and annealed powder are 5364 and 7053 (mW/cm^3^), respectively, a difference of 31.4%. 

The relative compacted density has the greatest influence on hysteresis loss. The main factors influencing eddy current loss are powder resistivity and particle size [6,17]. The most effective way to reduce eddy current loss is to increase resistivity, and there are two methods for doing so. The first is to add alloying elements like Si to the iron powder to increase resistivity, and the second is to form an insulating layer on the surface of the iron powder [18,19]. Because FeSiCr alloy powder has a higher resistivity than carbonyl iron powder, it should have a lower eddy current loss. However, the eddy current losses of carbonyl iron powder and FeSiCr alloy powder at 1 MHz are 1500 and 1600 (mW/cm^3^), respectively, and the high-frequency eddy current losses of the two powders are not significantly different. When the particle size of the magnetic powder increases, the eddy current loss increases. Because the particle size of FeSiCr alloy powder is larger than that of carbonyl iron powder, there is no significant difference in high-frequency eddy current loss between the two. The annealed powder has an eddy current loss of up to 2500 (mW/cm^3^) at 1 MHz, approximately 56% higher than the carbonyl iron powder. The main reason for this is that the particle size of the annealed powder is roughly twice that of the carbonyl iron powder, and the surface insulation layer may be damaged during the molding process due to the lower plastic deformation caused by the superior mechanical property of the annealed powder.

### 3.2. Binary Mixtures of Carbonyl Iron Powder, FeSiCr Alloy Powder, and Annealed Alloy Powder

Table 4 shows the compacted density, relative compacted density, and permeability obtained at a 50% mixed powder ratio. Adding 50% carbonyl iron powder to the annealed powder is particularly interesting, resulting in a relative compacted density of 93.2%. This value exceeds pure carbonyl iron powder and pure annealed powder, indicating that smaller carbonyl iron particles are more compressible and act as effective interstice fillers between the annealed powder particles. As a result, the mixed powder had a higher relative compacted density than the two individual base powders.

**Figure 3:**
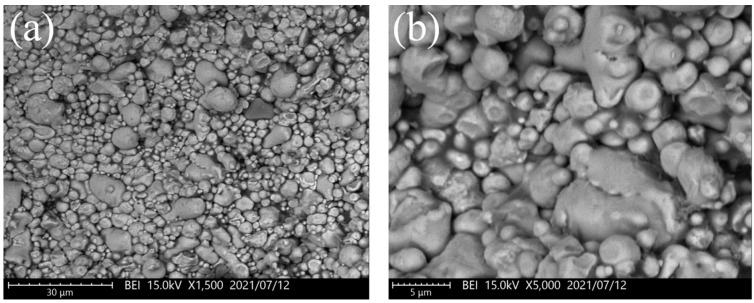

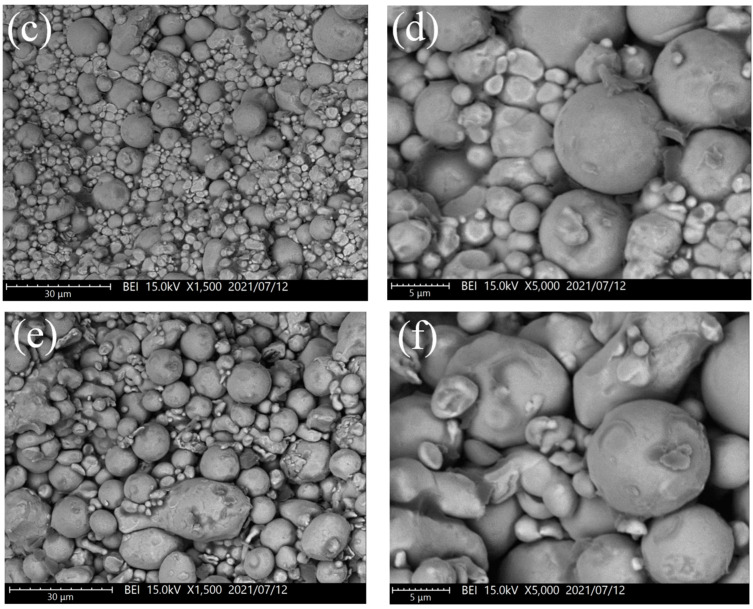
SEM images of the compacted samples at a 50% mixed powder ratio: (**a**,**b**) carbonyl iron powder and FeSiCr alloy powder mixture, (**c**,**d**) carbonyl iron powder and annealed powder mixture, and (**e**,**f**) FeSiCr alloy powder and annealed powder mixture.

Because the FeSiCr alloy powder is water-atomized, the particle morphology is irregular, resulting in non-uniform gaps between particles. As a result, it is difficult for the carbonyl iron powder to fill these interstices uniformly, resulting in a slight increase in relative compacted density from 87.0% to 88.9%. The FeSiCr alloy and the annealed powders have an average particle size of around 10 μm for mixtures of the FeSiCr alloy and the annealed powders, resulting in no interstice filling effect. Despite its lack of compressibility, the spherical annealed powder can easily rearrange to fill the gaps created during compaction. As a result, the relative compacted density exceeded that of pure alloy powder and annealed powder by 89.6%. The SEM images of the compacted samples at a 50% mixed powder ratio are shown in Figure 3. The mixture of the carbonyl iron powder and annealed powder had a denser microstructure because of the easy rearrangement caused by the spherical shape of both powders, the interstice filling, and the severe plastic deformation of carbonyl iron powder during compaction. On the other hand, the FeSiCr alloy powder added with annealed powder or carbonyl iron powder exhibited a less dense microstructure because the particle morphology of the FeSiCr alloy powder is irregular, resulting in non-uniform gaps between particles. This is consistent with the result of relative compacted density (Table 4).

Permeability follows the same trend as compaction density, with greater permeability resulting from higher relative compacted density. Adding 50 wt% carbonyl iron powder to the annealed powder yields the highest permeability (reaching 35.3), while the individual alloy powder has the highest permeability among the single powders, but only at 31.0. 

**Figure 4:**
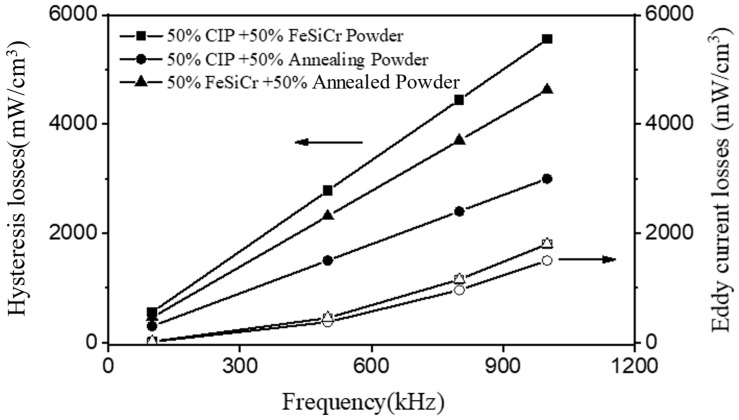
Variation of the eddy current loss (P_e_) and hysteresis loss (P_h_) with the measurement frequency for the toroidal cores made from the binary mixtures of carbonyl iron powder, FeSiCr alloy powder, and annealed alloy powder.

Figure 4 shows the variation of the eddy current loss (P_e_) and hysteresis loss (P_h_) with the measurement frequency for the toroidal cores made from the binary mixtures of carbonyl iron powder, FeSiCr alloy powder, and annealed alloy powder. It reveals that hysteresis loss in this frequency range primarily dominated the power loss. The hysteresis loss was lowest when 50 wt% carbonyl iron powder was added to the annealed powder. The primary reason is that it has the highest compacted density (fewest pores), allowing for easier movement of domain walls or magnetic domain rotation when subjected to an external magnetic field. It has a lower coercivity and, as a result, the lowest hysteresis loss. When 50 wt% FeSiCr alloy powder was added to the annealed powder, even though the FeSiCr alloy powder had larger particle sizes than carbonyl iron powder, the presence of numerous impurity elements impeded the movement of domain walls or magnetic domain rotation under the influence of an external magnetic field, resulting in a relative increase in hysteresis loss [16].

For the mixtures of carbonyl iron powder and FeSiCr alloy powder, both powders exhibited more severe plastic deformation during compaction, resulting in a significant number of dislocations. This abundance of dislocations hindered the movement of domain walls or magnetic domain rotation, combined with the lower relative compacted density, resulting in the highest hysteresis loss among the three mixtures, reaching 5557 (mW/cm^3^) at 1 MHz.

Regarding eddy current loss, the three mixtures exhibit the same trends as hysteresis loss. The carbonyl iron and annealed powder mixture had the lowest eddy current loss. When smaller-sized carbonyl iron powder was used as a filler for the spherical annealed powder, the insulating layer of the annealed powder was less likely to fracture during the compaction process. As a result, there was no significant decrease in electrical resistivity, resulting in the least amount of eddy current loss. In contrast, as larger-sized FeSiCr alloy powder was added to the annealed powder, interconnected FeSiCr alloy powders were more likely to form after compaction due to the larger particle size and severe plastic deformation of the FeSiCr alloy powder. Furthermore, the annealed powder’s insulating layer was prone to fracturing during compaction, resulting in lower electrical resistivity than cores made with a carbonyl iron powder and annealed powder. This significantly increased eddy current loss.

## 4. Conclusions

This study investigated the magnetic properties of soft magnetic composites (SMCs) made from carbonyl iron powder, FeSiCr alloy powder, and annealed FeSiAl alloy powder. The study shed light on the effect of different powder compositions on the density, microstructure, and magnetic properties of molding compaction inductors. The findings shed light on the design and optimization of soft magnetic composites for improved magnetic performance and lower power loss.

The findings revealed that hysteresis loss is the most significant contributor to power loss in the tested frequency range. Adding 50% carbonyl iron powder to annealed powder resulted in the lowest hysteresis loss, owing to its higher compacted density and lower coercivity, which facilitated domain wall movement. Incorporating 50% FeSiCr alloy powder with annealed powder, on the other hand, resulted in higher hysteresis loss due to impurity components impeding domain wall motion. Due to significant plastic deformation during compaction, the combination of carbonyl iron powder and FeSiCr alloy powder demonstrated the greatest hysteresis loss. 

Furthermore, the study looked at binary mixtures of these powders and discovered that adding carbonyl iron powder to annealed powder resulted in the highest relative compacted density, which led to improved magnetic properties. Among the mixtures, the combination of carbonyl iron powder and annealed powder had the lowest hysteresis and eddy current losses.

These discoveries are useful for improving the design of soft magnetic composites, particularly in the context of molded inductor manufacturing. The study advances magnetic materials and provides insight into developing high-performance inductor systems for electronic and electrical applications. As a result, the research has important implications for improving energy efficiency and lowering power loss in power systems and magnetic components. 

## Figures and Tables

**Table 1:** Particle size distribution, powder shape, true density, tape density, and chemical composition of the magnetic powders.

Powder Type	FeSiCr Alloy Powder	Carbonyl Iron Powder	Annealed Powder
Particle Size (µm)	D_10_ = 4.1, D_50_ = 9.4, D_90_ = 20.4	D_10_ = 2.5, D_50_ = 4.4, D_90_ = 6.9	D_10_ = 5.8, D_50_ = 9.1, D_90_ = 13.0
Powder Shape	Irregular	Nearly Spherical	Nearly Spherical
True Density (g/cc)	7.8	7.7	7.0
Tap Density (g/cc)	4.4	4.6	4.2
Chemical Compositions (wt%)	Fe: 91.8	Fe: >99.5	Fe: 86.5
	Cr: 4.5	C: 0.01	Si: 7.0
	Si: 3.5	O: 0.28	Al: 6.5

**Table 2:** Comparison of compacted densities, relative compacted densities, and initial permeabilities of carbonyl iron powder, FeSiCr alloy powder, and annealed alloy powder.

Sample	Compacted Density (Relative Compacted Density)	Permeability
Carbonyl iron powder	6.2 g/cm^3^ (92.4%)	27.3
FeSiCr alloy powder	5.9 g/cm^3^ (87.0%)	31.0
Annealed FeSiAl powder	4.9 g/cm^3^ (79.2%)	23.4

**Table 3:** Summary of the magnetic losses of the toroidal cores made of carbonyl iron powder, FeSiCr alloy powder, and annealed powder compacted at 600 MPa.

Powder	Carbonyl Iron Powder	FeSiCr Powder	Annealed Powder
Core losses (mW/cm^3^)			
100 kHz	534	630	666
500 kHz	3025	3476	4163
800 kHz	5289	5964	7250
1000 kHz	6817	7739	9531
Hysteresis losses (mW/cm^3^)			
100 kHz	536	619	705
500 kHz	2682	3095	3526
800 kHz	4291	4952	5642
1000 kHz	5364	6190	7053
Eddy current losses (mW/cm^3^)			
100 kHz	15	16	25
500 kHz	375	400	625
800 kHz	960	1024	1600
1000 kHz	1500	1600	2500

**Table 4:** Compacted density, relative compacted density, and permeability obtained at a 50% mixed powder ratio.

Sample	Compacted Density (Relative Compacted Densities)	Permeability
Carbonyl iron and FeSiCr alloy powders	6.0 g/cm^3^ (88.9%)	31.9
Carbonyl iron and annealed FeSiAl alloy powders	6.0 g/cm^3^ (93.2%)	35.3
FeSiCr and annealed FeSiAl alloy powders	5.8 g/cm^3^ (89.6%)	34.7

## Data Availability

The data presented in this study are available in the article.
